# P-1865. Immunotherapy with Nebulized Toll-Like Receptor Agonists Restores Severe Immune Paralysis and Improves Outcomes in Mice with Influenza-Associated Pulmonary Aspergillosis

**DOI:** 10.1093/ofid/ofae631.2026

**Published:** 2025-01-29

**Authors:** Sebastian Wurster, Jezreel Pantaleón García, Nathaniel D Albert, Keerthi Bhoda, Yongxing Wang, Dimitrios P Kontoyiannis, Scott Evans

**Affiliations:** The University of Texas MD Anderson Cancer Center, Houston, Texas; The University of Texas MD Anderson Cancer Center, Houston, Texas; The University of Texas MD Anderson Cancer Center, Houston, Texas; The University of Texas MD Anderson Cancer Center, Houston, Texas; The University of Texas MD Anderson Cancer Center, Houston, Texas; The University of Texas MD Anderson Cancer Center, Houston, Texas; The University of Texas MD Anderson Cancer Center, Houston, Texas

## Abstract

**Background:**

Given the poor outcomes of influenza-associated pulmonary aspergillosis (IAPA), there is a major unmet need for innovative therapeutic strategies. Impairment of pathogen recognition receptor (PRR) signaling is a known pathomechanism and prognostic factor in IAPA patients. Therefore, we performed immunotherapy with nebulized PRR agonists in a murine IAPA model and studied its impact on infection outcomes and pulmonary immunopathology.

**Figure:**
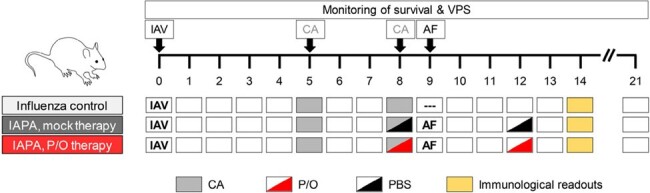

Timeline of experimental interventions. Abbreviations: AF = Aspergillus fumigatus, CA = cortisone acetate (10 mg intraperitoneally), IAV = nebulization with influenza A virus (7.5% of the 90% lethal dose), PBS = phosphate-buffered saline (mock therapy), P/O = Pam2/ODN, VPS = viral pneumonia score (0 = healthy – 12 = moribund).

**Methods:**

8-week-old BALB/c mice were infected with influenza A virus (IAV, H3N2), immunosuppressed with cortisone acetate (CA) on days (d) 5 and 8 after IAV infection, and infected intranasally with 50,000 *Aspergillus fumigatus* (AF) conidia on d9 (Fig. 1). Mice received nebulized Pam2+ODN (P/O, 4 µM/1 µM) or phosphate-buffered saline (mock therapy) before and after AF super-infection (d8/d12). Therapeutic outcomes were assessed using a combined morbidity (viral pneumonia score ≥ 7/12) and mortality endpoint. On d14, nCounter-based transcriptomics with Ingenuity pathway enrichment analysis was performed on lung tissue, along with histopathology and flow cytometry.

**Figure:**
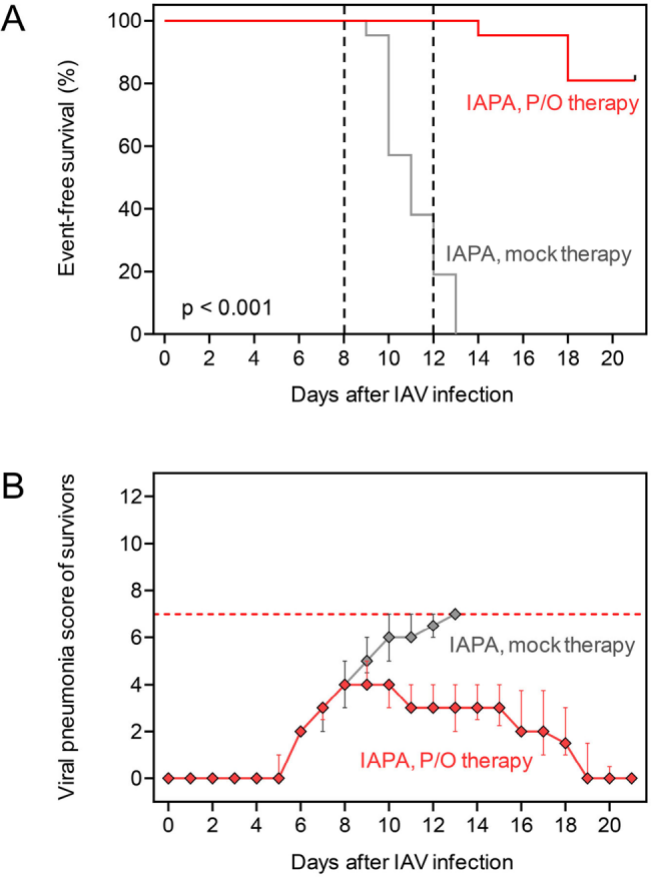

Event-free survival (A; Mantel-Cox log-rank test) and median viral pneumonia scores of survivors (B) depending on the treatment arm.

**Results:**

All mock-treated mice with IAPA reached the morbidity/mortality endpoint by d13 (Fig. 2). Compared to CA-immunosuppressed mice with IAV infection only (100% survival), nCounter analysis revealed minimal incremental inflammatory response and suppression of several PRR pathways, interferon signaling, and neutrophil activation in the IAPA group. P/O therapy led to 80% event-free survival until d21 (p < 0.001 vs. mock therapy) and full recovery of all survivors (Fig. 2). Histopathology revealed significantly reduced fungal burden and hemorrhagic lesions in P/O-treated animals. nCounter analysis showed induction of PRR signaling, several key effector cytokine pathways, and epithelial signaling after P/O therapy. Moreover, nCounter and flow cytometry revealed enhanced recruitment of macrophages, natural killer cells, and T cells in P/O-treated mice (Fig. 3).

**Figure:**
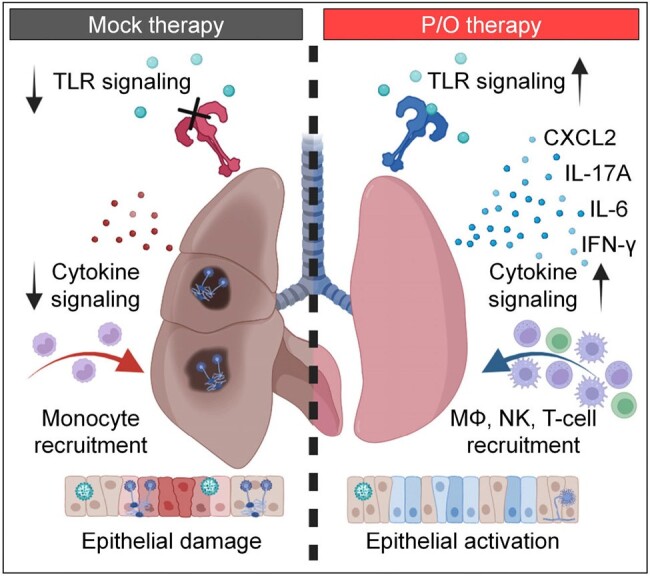

Summary of nCounter and flow cytometry results.

**Conclusion:**

Consistent with previous studies in human IAPA patients, our data revealed severe immune paralysis in mice with IAPA. Immunotherapy with nebulized P/O strongly improved infection outcomes, enhanced mobilization of key immune effector cells, and re-invigorated PRR and cytokine signaling.

**Disclosures:**

Sebastian Wurster, MD, MSc, Astellas Pharma: Grant/Research Support|Gilead Sciences: Grant/Research Support Dimitrios P. Kontoyiannis, MD, AbbVie: Advisor/Consultant|Astellas Pharma: Advisor/Consultant|Astellas Pharma: Grant/Research Support|Astellas Pharma: Honoraria|Cidara Therapeutics: Advisor/Consultant|Gilead Sciences: Advisor/Consultant|Gilead Sciences: Grant/Research Support|Gilead Sciences: Honoraria|Knight: Advisor/Consultant|Merck: Advisor/Consultant|Scynexis: Advisor/Consultant

